# The utility of quantifying the orientation of breast masses in ultrasound imaging

**DOI:** 10.1038/s41598-024-55298-w

**Published:** 2024-02-25

**Authors:** Kailiang Chen, Size Wu

**Affiliations:** Department of Ultrasound, The First Affiliated Hospital of Hainan Medical University, No.31, Longhua Road, Haikou, 570102 China

**Keywords:** Breast mass, Malignancy, Breast imaging-reporting and data system (BI-RADS), Orientation, Angle, Cancer, Diseases, Health care, Oncology

## Abstract

The aim of this study was to quantify the orientation of breast masses and determine whether it can enhance the utility of a not parallel orientation in predicting breast mass malignancy. A total of 15,746 subjects who underwent breast ultrasound examinations were initially enrolled in the study. Further evaluation was performed on subjects with solid breast masses (≤ 5 cm) intended for surgical resection and/or biopsy. The orientation angle, defined as the acute angle between the align of the maximal longitudinal diameter of the breast mass and the surface of the breast skin, was measured. Receiver operating characteristic (ROC) curve analysis was conducted, and various performance measures including sensitivity, specificity, positive and negative predictive values, accuracy, odds ratio, and the area under the ROC curve (AUC) were calculated. Multivariate analysis was performed to determine if the orientation angle was an independent predictor of breast malignancy. Decision curve analysis (DCA) was also conducted to assess the net benefit of adopting the orientation angle for predicting breast mass malignancy. The final analysis included 83 subjects with breast cancer and 135 subjects with benign masses. The intra-group correlation coefficient for the measurement of the orientation angle of breast masses was 0.986 (*P* = 0.001), indicating high reproducibility. The orientation angles of malignant and benign breast masses were 36.51 ± 14.90 (range: 10.7–88.6) degrees and 15.28 ± 8.40 (range: 0.0–58.7) degrees, respectively, and there was a significant difference between them (*P* < 0.001). The cutoff value for the orientation angle was determined to be 22.9°. The sensitivity, specificity, positive and negative predictive values, accuracy, odds ratio, and AUC for the prediction of breast malignancy using the orientation angle were 88.0%, 87.4%, 81.1%, 92.2%, 87.6%, 50.67%, and 0.925%, respectively. Multivariate analysis revealed that the orientation angle (> 22.9°), not circumscribed margin, and calcifications of the breast mass were independent factors predicting breast malignancy. The net benefit of adopting the orientation angle for predicting breast malignancy was 0.303. Based on these findings, it can be concluded that quantifying the orientation angle of breast masses is useful in predicting breast malignancy, as it demonstrates high sensitivity, specificity, AUC, and standardized net benefit. It optimizes the utility of the not parallel orientation in assessing breast mass malignancy.

## Introduction

Breast cancer was the leading cause of global cancer incidence in 2020, representing 11.7% of all cancer cases^1^. It is the fifth leading cause of cancer mortality worldwide^1^. Among women, breast cancer accounts for one in four cancer cases and one in six cancer deaths, ranking first for incidence in most countries (159 out of 185 countries) and for mortality in 110 countries^1^. Currently, there is no effective way to prevent breast cancer, and early detection of breast lesions through screening and effective treatment are the best methods to reduce breast cancer mortality^2–5^.

Mammogram is the conventional modality for breast cancer screening; however, it has limitations in dense breasts, which may lead to overdiagnosis^6^. Up-to-date, the American College of Radiology recommends that for most women at higher-than-average risk of breast cancer and women with dense breasts, the supplemental screening method of choice is breast magnetic resonance imaging (MRI). For those who cannot undergo breast MRI, contrast-enhanced mammography or ultrasound (US) could be considered^7^. MRI is a time-consuming, high-cost, and less accessible modality, which limits its application in a large population. Contrast-enhanced mammography is less expensive and more accessible than MRI and has the potential to display abnormal morphologic features and breast cancer-associated neovascularity, with high sensitivity approaching that of MRI^8^. On the other hand, it has drawbacks such as a slightly increased radiation dose compared to conventional mammography and a low risk for contrast allergy reactions^8^. US is a widely available, noninvasive, convenient, and cost-effective modality that is suitable for breast screening and diagnosis in a large population. Conventional US can detect and characterize breast masses and non-mass lesions and provide information for predicting the risk of malignancy^9–13^. The malignant breast mass is usually stiffer than a benign mass. US elastography can measure the stiffness of breast masses and provide useful parameters for breast evaluation^14,15^. Breast cancer and benign breast masses have different morphological features and distribution of the vascularity, and contrast-enhanced US can characterize these features and provide information for studying malignancy risk^16,17^. Deep learning and artificial intelligence have offered a new way to improve the prediction of malignancy risk based on breast US^18,19^. Despite the progress, the features of breast masses acquired by conventional gray-scale US are still essential, so further study on the US features and associated data of breast masses is still relevant. Several studies have assessed the value of US features and Breast Imaging-Reporting and Data System (BI-RADS) descriptors in differentiating between benign and malignant breast masses^10,11^. For example, Hong et al. and Gu et al. reported positive predictive values of 86%, 62%, and 69%, and 83.7%, 57.2%, and 63.9%, respectively, for predicting malignancy using US features such as a spiculated margin, an irregular shape, and a not parallel orientation (taller than wide shape)^10,11^.

A not parallel orientation of a breast mass is an important feature that affects malignancy risk and has been confirmed in numerous studies^8,10,11,20–22^. The growth orientation of breast masses receives significant attention in clinical practice when using the BI-RADS ultrasound lexicon descriptor^9–11,20^. The term "parallel" refers to lines extending in the same direction, equidistant from each other, and not meeting. "Not parallel" indicates lines extending in the same direction, with distinct distances and meeting at a point. Alternatively, parallel indicates a zero-degree angle, while nonparallel means an angle greater than 0° and less than 180°. Wang et al. discovered that breast cancer masses with a vertical orientation had a poor prognosis, suggesting that identifying the growth orientation of breast cancer may help predict its prognosis^23^. A parallel or not parallel orientation of a breast mass is based on a simple visual impression, and a parallel orientation may not truly be parallel. Not parallel can be arbitrarily defined with a broad range of degrees, resulting in subjective and inaccurate results, which may introduce bias or error in subsequent dichotomy analysis. This dichotomy is a broad categorization, as there is no specific threshold value for defining parallel and not parallel orientations, and accurate quantitative criteria have not been established. It is still unknown whether determining the orientation can optimize the prediction of malignancy. Therefore, in this study, we hypothesized that quantifying the orientation of breast masses may enhance the interpretation and utility of a not parallel orientation and improve the prediction of breast mass malignancy.

## Materials and methods

### Study population

**Figure 1:**
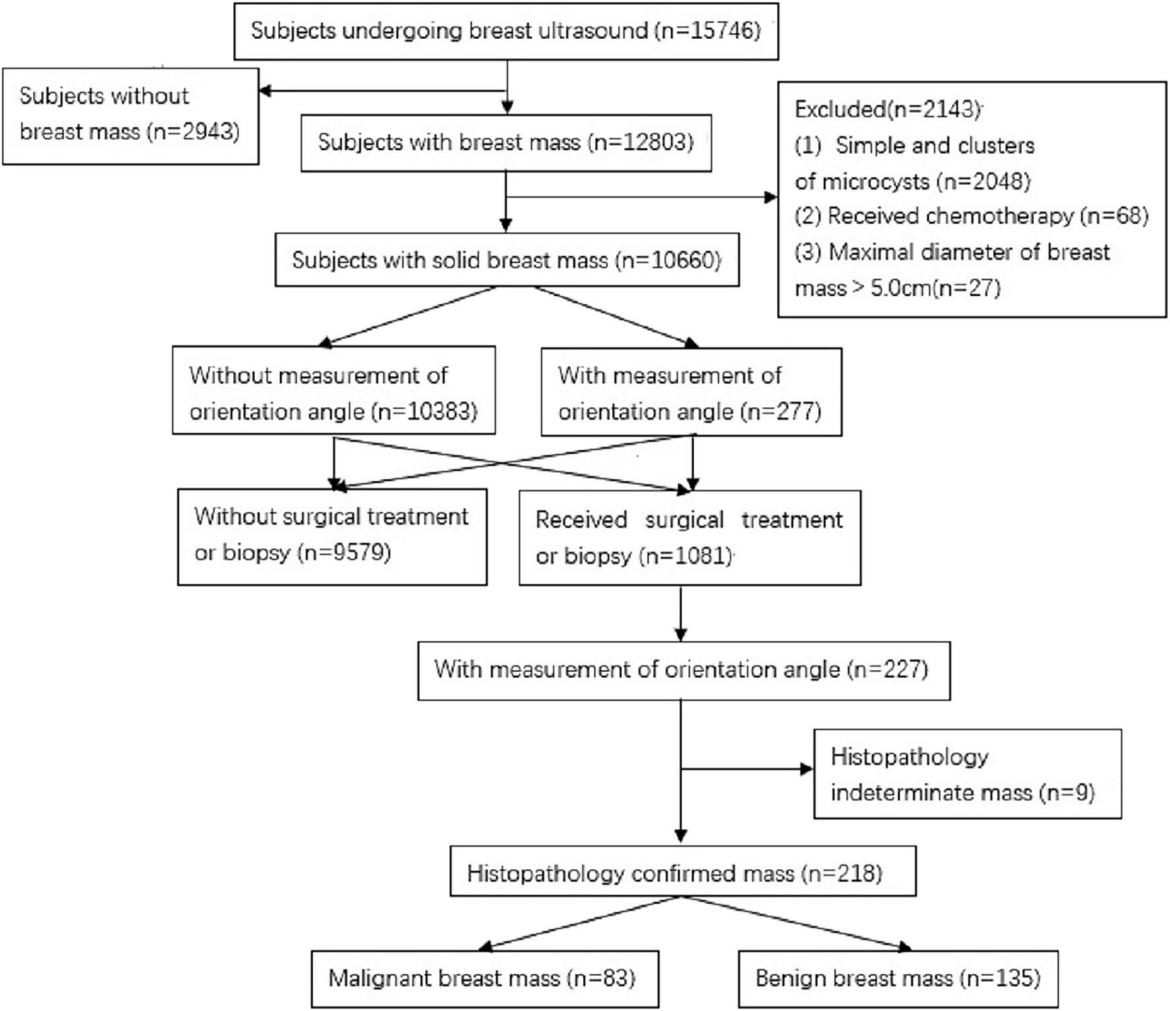
The flowchart shows the reasons subjects were excluded from this study.

This prospective study was conducted at The First Affiliated Hospital of Hainan Medical University from March 2022 to January 2023. A total of 15,746 subjects who underwent breast US screening examinations or presented with breast pain and received US examinations were considered for inclusion. The inclusion criteria were: (1) female subjects, (2) subjects aged 18–90 years, and (3) subjects who provided informed consent. The exclusion criteria were: (1) male subjects, (2) subjects with thoracic malformation, (3) subjects who had received chemotherapy for the breast mass, (4) subjects who were pregnant or breastfeeding, (5) subjects with non-mass lesions and breast lesions in which the lower margin was not clearly visible on sonography, and (6) subjects with inflammatory breast cancer. The flow chart of the initial subject enrollment is depicted in Fig. 1. The breasts were evaluated using the fifth edition of the BI-RADS US Atlas, and US descriptors such as shape, orientation, margin, echo pattern, posterior features, calcifications in mass (outside of a mass), intraductal calcifications, architectural distortion, etc., were used for description and image interpretation^9^. During the US examination, if multiple masses were identified in a subject, only the mass that was most suspicious of harboring malignancy or the representative mass with benign features identified during the US examination was selected.

### Ethical approval

This study was approved by the Ethics Review Committee of The First Affiliated Hospital of Hainan Medical University [2022 (Scientific Research) (No. 84)], and it was conducted in accordance with the Declaration of Helsinki. Written informed consent was obtained from all subjects.

### Image acquisition and measurements of the orientation angle of the breast mass

**Figure 2:**
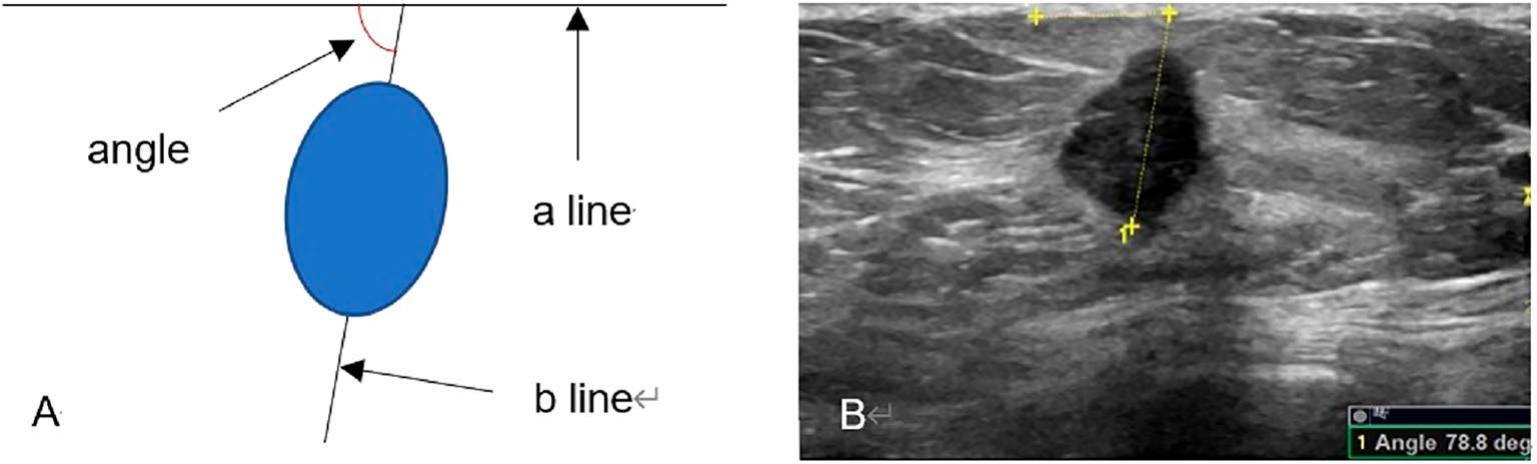
Measurement of the orientation angle. (**A**) The left plot is a schematic diagram illustrating the measurement of the orientation angle. Line a represents the interface between the skin of the breast and a transducer placed perpendicular to the skin of the breast. Line b represents the maximal diameter of the breast mass and its extension. The orientation angle is the acute angle between line a and line b. (**B**) The right plot shows an actual measurement of the orientation angle (78.8°) in a subject with breast cancer.

**Figure 3:**
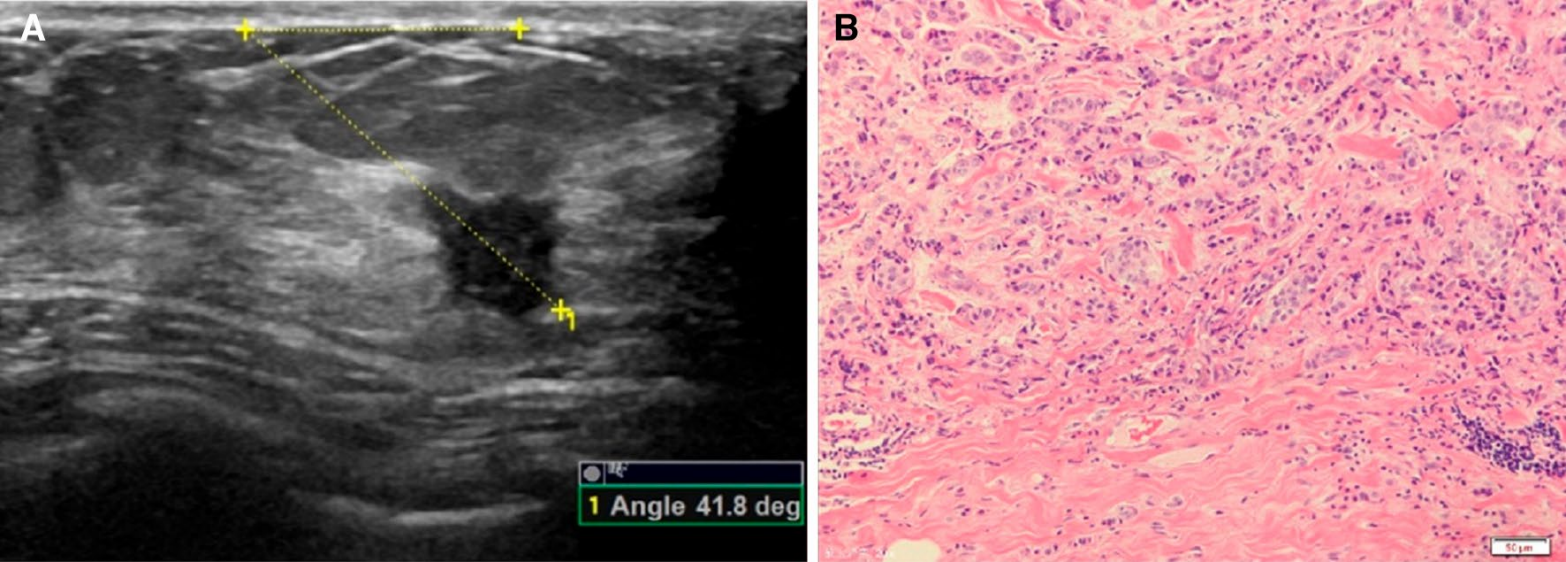
A 37-year-old woman with ductal infiltrating carcinoma in the right breast. (**A**) The left plot shows the orientation angle of 41.8°. (**B**) The right plot shows the histopathology (HE × 200).

**Figure 4:**
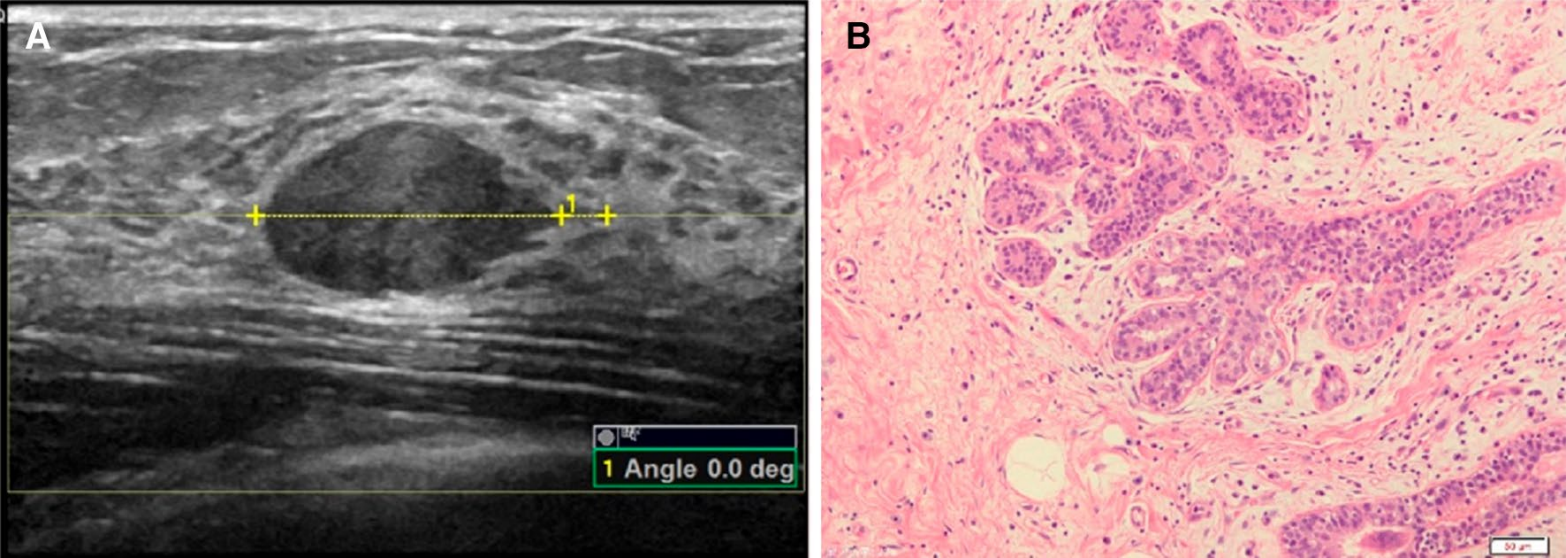
A 30-year-old woman with adenosis and adenomatous hyperplasia in the left breast. (**A**) The left plot shows the orientation angle of 0.0°. (**B**) The right plot shows the histopathology (HE × 200).

**Figure 5:**
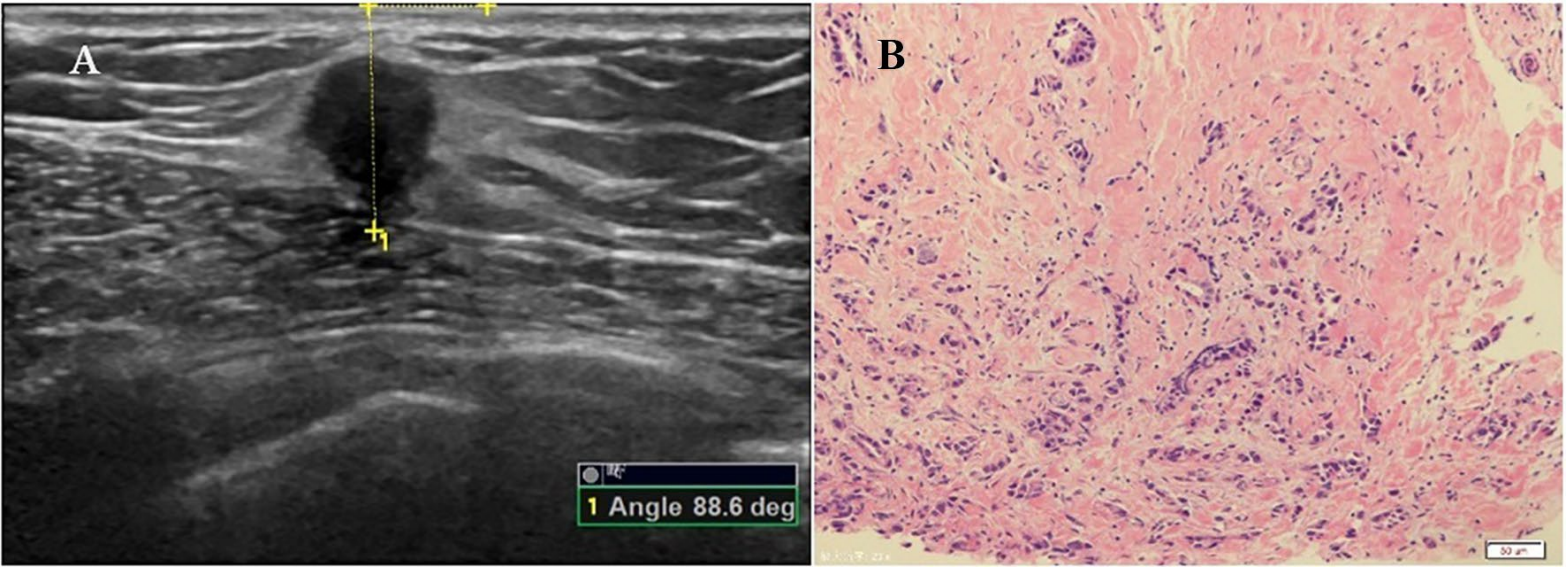
A 62-year-old woman with invasive carcinoma in the left breast. (**A**) The left plot shows the orientation angle of 88.6°. (**B**) The right plot shows the histopathology (HE × 200).

Breast US examinations were conducted using Mindray Resona7 (Shenzhen Mindray Bio-Medical Electronics Co., Ltd., Shenzhen, China) and Logiq E9 (General Electric Healthcare, Milwaukee, WI, USA) ultrasound systems. The systems were adjusted to small parts mode (breast), and linear array transducers with a frequency of 5–15 Mega Hertz (MHz) were used. The subjects were positioned in a supine position on a plain table without a pillow under the head. The upper limbs were abducted at a right angle to the trunk, with the forearms flexed at a right angle to the upper arms to fully expose the breast and axillary regions. A thorough scan of the entire breast and axillary regions was performed to screen for any masses. The US features of the mass and any associated features were described according to the BI-RADS US lexicon. Color Doppler flow imaging was then performed by a radiologist specialized in US to assess the vascularity of the breast mass. The breast masses were categorized by two radiologists specialized in US, following the BI-RADS guidelines. The categories included: category 1 (negative findings), category 2 (benign findings), category 3 (probably benign findings), category 4 (findings suspicious for malignancy), and category 5 (findings highly suggestive of malignancy). For subjects with solid breast masses measuring ≤ 5 cm and intended for surgical resection and/or biopsy, further evaluation of the orientation was conducted by measuring an orientation angle. The acute angle between the maximal longitudinal diameter of the breast mass at its greatest dimension and the surface of the breast skin was measured using the built-in software of the US system, as illustrated in Figs. 2, 3, 4 and 5. Adequate coupling gel was applied to the breast skin, and the transducer was gently placed on the breast skin without applying pressure. The measurement of the orientation angle was performed by a radiologist specialized in US with 14 years of experience in breast imaging using the General Electric Logiq E9 US system. The intra-group correlation coefficient (ICC) of the measurement of the orientation angle was determined at the beginning of the study by measuring the orientation angle twice in 30 subjects who were consecutively enrolled from the subjects referring for breast mass US examination. Representative images were saved in the Picture Archiving and Communication Systems. The distribution of orientation angles for malignant and benign breast masses was calculated, and a cutoff value was determined to predict breast mass malignancy.

### Statistical analysis

The age of the subjects and the sizes of the breast masses, which followed a normal distribution, were expressed as mean ± standard deviation. The measurements of the orientation angle, which did not follow a normal distribution, were expressed as median and interquartile range. Categorical variables were presented as percentages. One-way analysis of variance was used to analyze the normal distributed continuous variables of age, breast mass size, and BMI. Chi-square test or Fisher’s exact test was used to analyze the categorical variables of study sample characteristics. ICC as calculated to assess the consistency of the measurements of the orientation angle. Comparison among the distributions of the orientation angles of the breast masses in different BI-RADS categories was performed using one-way analysis of variance, and the correlation between them was calculated. The pathological results were considered as the diagnostic gold standard. Receiver operating characteristic (ROC) curve analysis was conducted, and the area under the ROC curve (AUC) was calculated. The cut-off value of the orientation angle was determined based on the Youden index. The sensitivity, specificity, positive and negative predictive values, accuracy, and odds ratio were calculated. Multivariate analysis was performed to investigate whether the orientation angle was an independent predictor for breast malignancy, considering other US characteristics such as shape, margin, echo pattern, posterior features, and calcifications. Decision curve analysis (DCA) was conducted using R for Windows 3.6.1 to determine the net benefit of adopting the orientation angle. Statistical Product and Service Solutions (SPSS) 26.00 software was used for other statistical analyses. The level of statistical significance was set at *P* < 0.05 (two-tailed).

## Results

### Final sample of the study

The study included a total of 15,746 subjects who underwent US examination. Out of these, 12,803 subjects were found to have breast focal lesions (masses). Among the 12,803 subjects with breast masses, 10,660 had solid masses. A total of 1081 subjects underwent surgical resection and/or biopsy. Among the subjects with breast masses, 277 subjects had measurements of the orientation angle of the breast mass. Histopathology results were available for 227 patients with 227 breast masses. These included 83 malignant masses, 135 benign masses, and 9 masses with indeterminate pathology. Some subjects were excluded from the study. This included 2048 subjects with simple and clusters of microcysts, 68 subjects who had undergone chemotherapy for the breast mass, and 27 patients with a maximum lesion diameter larger than 5 cm. The demographic and baseline characteristics of the study subjects are summarized in Table 1. A flow chart illustrating the subject enrollment and investigation process is shown in Fig. 1. The pathologies and distribution of the breast lesions are summarized in Table 2.

**Table 1:** Study sample characteristics.

Characteristic	Malignant mass (n = 83)	Benign mass (n = 135)	*P* value
Long diameter (mm)	20.43 ± 8.96(7.5–46.4)*	18.32 ± 7.52(7.5–47.8)*	0.075
Short diameter (mm)	13.06 ± 6.24(2.1–28.0)*	9.09 ± 4.26(3.4–28.9)*	0.001
Age (year)	51.27 ± 9.90 (31–71)*	36.64 ± 10.33 (18–60)*	0.001
BMI (kg/m^2^)	23.92 ± 3.08 (18.3–34.2)*	22.04 ± 3.04 (16.7–33.8)*	0.001
Family history of breast cancer			0.429
Yes	4 (4.9)	2 (1.8)	
No	77 (95.1)	107 (98.2)	
Breast pain			0.543
Yes	15 (18.1)	29 (21.5)	
No	68 (81.9)	106 (78.5)	
Nipple discharge			0.483
Yes	4 (4.8)	4 (3.0)	
No	79 (95.2)	131 (97.0)	
Palpable mass			0.001
Yes	73 (88.0)	71 (52.6)	
No	10 (12.0)	64 (47.4)	
Architectural changes			0.316
Skin thickening	3 (3.6)	1 (0.7)	
Architectural distortion	4 (4.8)	7 (5.2)	
No change	76 (91.6)	127 (94.1)	
Position of mass			0.369
Left	36 (43.4)	67 (49.6)	
Right	47 (56.6)	68 (50.4)	
Quadrant			0.095
Upper outer	38 (45.8)	60 (44.4)	
Upper inner	25 (30.1)	26 (19.3)	
Lower inner	5 (6.0)	20 (14.8)	
Lower outer	15 (18.1)	29 (21.5)	
Enlarged lymph nodes in axillary regions			0.001
Yes	22 (26.5)	6 (4.4)	
No	61 (73.5)	129 (95.6)	

Unless otherwise indicated, data are counts with percentages in parentheses; mean data are ± standard deviation. *Data in parentheses are ranges. BMI, Body mass index.

**Table 2:** Distribution of the breast lesions.

Pathology	Number (%)
Benign lesion	
Fibroadenoma	40 (29.7)
Adenosis with fibroadenoma like hyperplasia	47 (34.8)
Adenosis	34 (25.2)
Chronic granulomatous inflammation	5 (3.7)
Intraductal papilloma	4 (3.0)
Benign phyllodes tumor	2 (1.5)
Tubular adenoma	1 (0.7)
Solitary fibrous tumor	1 (0.7)
Total	135 (100.00)
Malignant lesion	
Non-invasive carcinoma	13 (15.7)
Invasive ductal carcinoma	44 (53.0)
Invasive carcinoma of special type	26 (31.3)
Total	83 (100.00)

Data are numbers of breast lesions and numbers in parentheses are percentages.

### Outcome of statistical analysis

**Figure 6:**
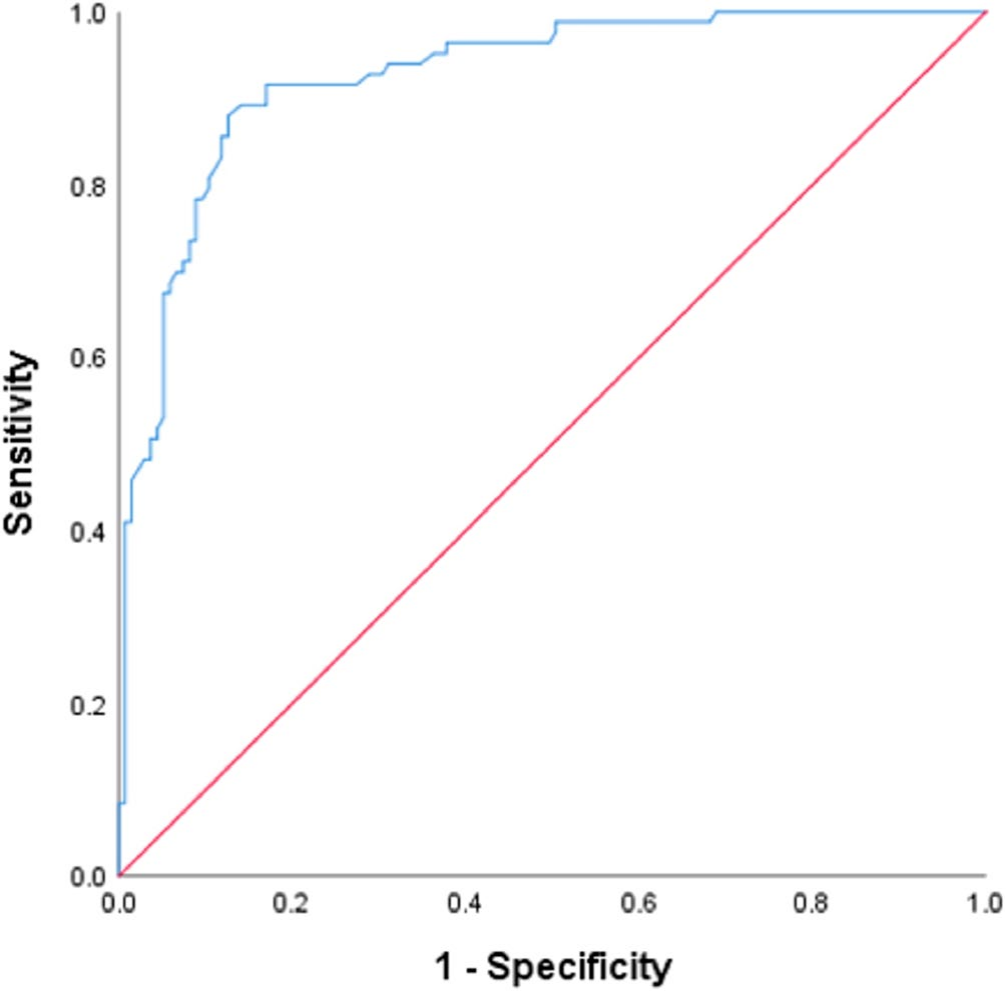
The receiver operating characteristic curve of the orientation angle for the assessment of the malignancy risk of breast masses.

**Figure 7:**
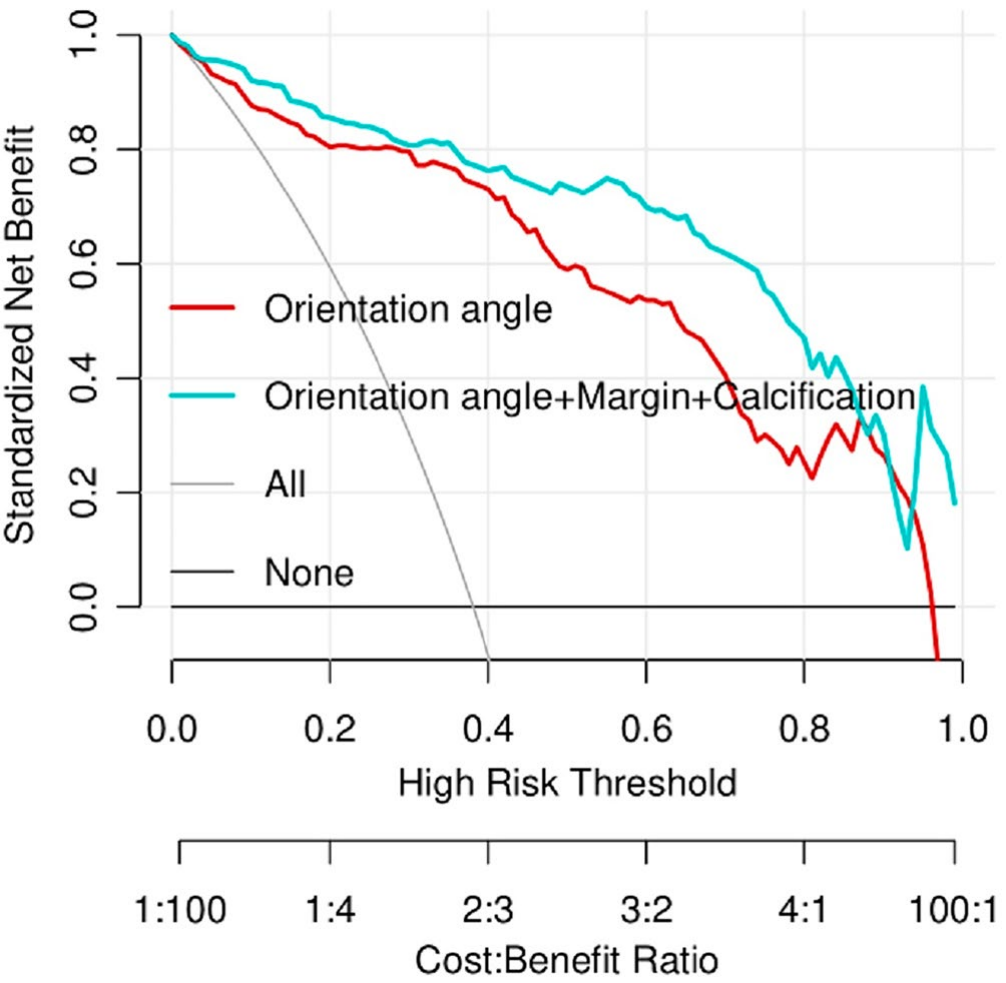
The decision curve analysis of the orientation angle and a combination of independent predictors for the assessment of the likelihood of breast malignancy.

The ICC for the measurement of the orientation angle of the breast mass was 0.986 (*P* = 0.001), indicating a high level of consistency. The maximum longitudinal diameters of malignant and benign breast masses were 1.83 ± 0.75 (range: 0.75–4.78) cm and 2.04 ± 0.89 (range: 0.75–4.64) cm, respectively. There was no significant difference between the two groups (*P* = 0.075, 95% confidence interval (CI) − 0.444 to 0.021). The orientation angles of malignant and benign breast masses were 36.51 ± 14.90 (range: 10.7–88.6) degrees and 15.28 ± 8.40 (range: 0.0–58.7) degrees, respectively. There was a significant difference between the two groups (*P* < 0.001, 95% CI − 24.769 to − 17.683). The differences in orientation angles of breast masses between BI-RADS category 3 and 4a, 4b, 4c, and 5, as well as between BI-RADS category 4a and 4b, 4c, and 5, were all significant (all *P* < 0.05). However, the differences in orientation angles of breast masses between BI-RADS category 4b and 4c, and 5, as well as between BI-RADS category 4c and 5, were not significant (all *P* > 0.05). The correlation between the orientation angle and BI-RADS category was 0.735 (*P* < 0.001), as shown in Table 3. The ROC curve of the orientation angle for assessing the malignancy risk of breast masses is presented in Fig. 6. The Youden Index was 0.754, and the cutoff value of the orientation angle was determined to be 22.9°. Among the breast masses, 41.3% (90/218) had an orientation angle > 22.9°, of which 81.1% (73/90) were malignant and 18.9% (17/90) were benign. On the other hand, 58.7% (128/218) of the breast masses had an orientation angle ≤ 22.9°, of which 92.2% (118/128) were benign and 7.8% (10/128) were malignant. The sensitivity, specificity, positive and negative predictive values, accuracy, odds ratio, and AUC for the prediction of breast malignancy were 88.0%, 87.4%, 81.1%, 92.2%, 87.6%, 50.67%, and 0.925% (*P* < 0.001, 95% CI 0.890–0.961), respectively. Multivariate analysis revealed that the orientation angle (> 22.9°), as well as the presence of a non-circumscribed margin and calcifications of the breast mass, were independent factors predicting breast mass malignancy. The orientation angle (> 22.9°) exhibited a net benefit of 0.303 for the prediction of breast mass malignancy, as shown in Fig. 7.

**Table 3:** Distribution of orientation angle of breast mass in BI-RADS 3, 4 and 5.

BI-RADS category	Number (%)	Orientation angle (°)
3	102 (46.79)	13.3 (8.6–19.0)
4a	35 (16.06)	18.6 (12.7–26.5)
4b	23 (10.55)	32.3 (24.6–44.9)
4c	29 (13.30)	33.9 (27.2–46.8)
5	29 (13.30)	32.6 (26.5–51.6)
Total	218 (100.00)	

Unless otherwise indicated, data in paratheses of the left column are percentage; and data in the right column are the median, with the interquartile range in parentheses.

## Discussion

The assessment of the likelihood of malignancy of a breast mass using US is primarily based on the US characteristics of the shape, orientation, margin, echo pattern, calcifications, and posterior features. These characteristics may not all be present in the same breast mass at the same time, and the importance of each characteristic varies. Parallel and not parallel orientations are associated mainly with benign and malignant breast tumors, respectively^8–11^. In this study, multivariate analysis showed that an orientation angle greater than 22.9°, a not circumscribed margin, and calcifications were independent factors predicting breast mass malignancy. These findings were consistent with previous studies, except that the not parallel orientation was replaced with the orientation angle greater than 22.9°^8–11,23^. The orientation angle of the breast mass was precisely measured in our investigation, using an objective and scientific process. The difference in orientation angle range between malignant and benign breast masses was significant, and the orientation angles increased with increasing malignancy risk in BI-RADS categories 3, 4a, 4b, 4c, and 5. There was a strong correlation between the orientation angle and BI-RADS, indicating that the orientation angle is associated well with the BI-RADS and has the potential to be integrated into it. Malignant breast masses typically had larger orientation angles, with a trend for not parallel orientation, while benign breast masses typically had smaller orientation angles with a trend for parallel orientation. These findings were consistent with previous studies^8,10,11,20–26^. However, there was some overlap between the orientation angles of benign and malignant breast masses, which may affect the differentiation of these two types of masses. When the cutoff value for the orientation angle was set at 22.9°, a sensitivity of 88.0%, a specificity of 87.4%, an AUC of 0.925, and an accuracy of 87.6% were obtained for the assessment of breast masses. Compared to a previous study that used a "taller than wide" orientation for predicting breast malignancy, the orientation angle of 22.9° demonstrated a substantially higher sensitivity and a comparable accuracy, indicating that it has better predictive ability. The odds ratio obtained in our study was also substantially higher than that reported in the previous study, further supporting the stronger association of the quantified orientation angle cutoff with breast malignancy. Overall, the assessment of the likelihood of malignancy of a breast mass using ultrasonography can be improved by considering the orientation angle as an independent factor. This angle provides valuable information and has better predictive ability compared to the traditional "taller than wide" orientation method.

In our study, the positive predictive value of 81.1% was almost the same as that obtained based on a "taller than wide" orientation reported by Stavros et al.^8^ (81.2%). However, it was much higher than the positive predictive value obtained by other studies that used a gross dichotomy analysis of the US BI-RADS descriptor of a not parallel orientation (ranging from 63.9 to 69%)^10,11,26^. This suggests that the established orientation angle has a greater ability to predict breast malignancy than the traditional method. The ICC of measurement for the orientation angle was 0.986, indicating that the measuring performance is highly reproducible, reliable, and appropriate. The good net benefit suggests that the orientation angle, either alone or combined with other independent predictors, may be more useful for assessing the likelihood of breast malignancy.

There were some limitations in this study: (1) This was a single-center study, and the subjects were of Han ethnic Chinese, which may have introduced a sample selection bias; and (2) The sample size was relatively small, resulting in the exclusion of many breast masses in lower BI-RADS categories due to the lack of histopathological results, which may have caused a sample enrollment bias. In the future, it is important to validate our study results in different study populations to assess their generalizability.

## Conclusions

Establishing the optimal orientation angle for the assessment of breast masses is beneficial in improving the positive predictive value of the BI-RADS ultrasound lexicon descriptor for breast mass malignancy. It provides high values of sensitivity, specificity, AUC, and a good net benefit, offering a new perspective for evaluating the risk of malignancy in breast masses.

### Supplementary Information

Supplementary Information.

## Data Availability

The data of the text were included in the Supplementary Information, and the images can be obtained from the authors (S.W.) upon reasonable request.
